# Prenatal Hypoxia and Placental Oxidative Stress: Insights from Animal Models to Clinical Evidences

**DOI:** 10.3390/antiox9050414

**Published:** 2020-05-12

**Authors:** Serena Silvestro, Valeria Calcaterra, Gloria Pelizzo, Placido Bramanti, Emanuela Mazzon

**Affiliations:** 1 Departmnent of Experimental Neurology, IRCCS Centro Neurolesi “Bonino-Pulejo”, Via Provinciale Palermo, Contrada Casazza, 98124 Messina, Italy; serena.silvestro@irccsme.it (S.S.); placido.bramanti@irccsme.it (P.B.); 2 Pediatric and Adolescent Unit, Department of Internal Medicine, University of Pavia, 27100 Pavia, Italy; valeria.calcaterra@unipv.it; 3 Department of Biomedical and Clinical Science “L. Sacco”, and Pediatric Surgery Department “V. Buzzi” Children’s Hospital, University of Milano, 20100 Milano, Italy; gloria.pelizzo@unimi.it

**Keywords:** prenatal hypoxia, reactive oxygen species, oxidative stress, animal models, clinical evidences

## Abstract

Hypoxia is a common form of intrauterine stress characterized by exposure to low oxygen concentrations. Gestational hypoxia is associated with the generation of reactive oxygen species. Increase in oxidative stress is responsible for damage to proteins, lipids and DNA with consequent impairment of normal cellular functions. The purpose of this review is to propose a summary of preclinical and clinical evidences designed to outline the correlation between fetal hypoxia and oxidative stress. The results of the studies described show that increases of oxidative stress in the placenta is responsible for changes in fetal development. Specifically, oxidative stress plays a key role in vascular, cardiac and neurological disease and reproductive function dysfunctions. Moreover, the different finding suggests that the prenatal hypoxia-induced oxidative stress is associated with pregnancy complications, responsible for changes in fetal programming. In this way, fetal hypoxia predisposes the offspring to congenital anomalies and chronic diseases in future life. Several antioxidant agents, such as melatonin, erythropoietin, vitamin C, resveratrol and hydrogen, shown potential protective effects in prenatal hypoxia. However, future investigations will be needed to allow the implementation of these antioxidants in clinical practice for the promotion of health in early intrauterine life, in fetuses and children.

## 1. Introduction

Fetal hypoxia is a condition characterized by a reduction of oxygen responsible for impairing the fetus development and increases the risk of perinatal and infant mortality [1]. Indeed, fetal hypoxia represents 23% of neonatal deaths worldwide [2]. The most common risk factors causing fetal hypoxia are placental insufficiency, preclampsia, umbilical cord damage and maternal factors such as smoking, heart, kidney or lung dysfunction [3].

Prenatal hypoxia can be classified into three patterns: pre-placental; uteroplacental and post-placental. The pre-placental hypoxia affects the fetus and the mother, unlike post-placental hypoxia that induces only fetal damage. Instead, uteroplacental hypoxia is hallmarked by an altered uteroplacental circulation [1]. We will focus on the last two patterns that are mainly related to the direct impact of hypoxia on the fetus.

Fetal hypoxia is responsible for the production of reactive oxygen species (ROS). ROS are essential to perform several cellular functions. However, excessive production of ROS cannot be countered by the antioxidant defenses; in this way, ROS are responsible for cellular oxidative stress. In the placenta, DNA damage, protein denaturation and lipid peroxidation induced by ROS can modify placental function leading to a reduction in oxygen and nutrients in the fetus [4]. This cascade of events induces limited fetal growth leading to a decrease in weight and a body shape alteration of the offspring [5]. Moreover, growth restriction is often correlated to abnormal organ development. This involves the birth of offspring with organs that may not support proper functioning during future life [6].

Specifically, exposure to short periods of fetal hypoxia leads to a redistribution of blood flow towards the essential circulation to protect the brain and heart [7]. However, prolonged periods of intrauterine hypoxia could entail alterations of the initial homeostatic defenses [8,9]. This implicates a high strain on the developing heart and principal vessels, inducing impaired fetal heart function. In this way, the bodyweight/heart weight ratio increases and occur changes in cardiac gene expressions [10]. Therefore, fetal hypoxia, due to alterations of fetal programming, represents an immediate danger to fetal life, but also to the future life [9]. Indeed, the exposure of the fetus to hypoxic conditions predisposes the offspring to congenital anomalies and chronic diseases, such as cardiovascular, renal and metabolic disorders, in later life [3,11,12,13,14,15,16].

Therefore, therapeutic interventions during pregnancy represent an interesting opportunity aimed to reduce the harmful effects of fetal hypoxia-induced oxidative stress.

In this review, we want to provide an overview of preclinical and clinical evidences aimed to illustrate the correlation between fetal hypoxia and oxidative stress. The prevention of the oxidative process of a long fetal life has to be considered to promote healthcare in children.

## 2. Methodology

**Figure 1:**
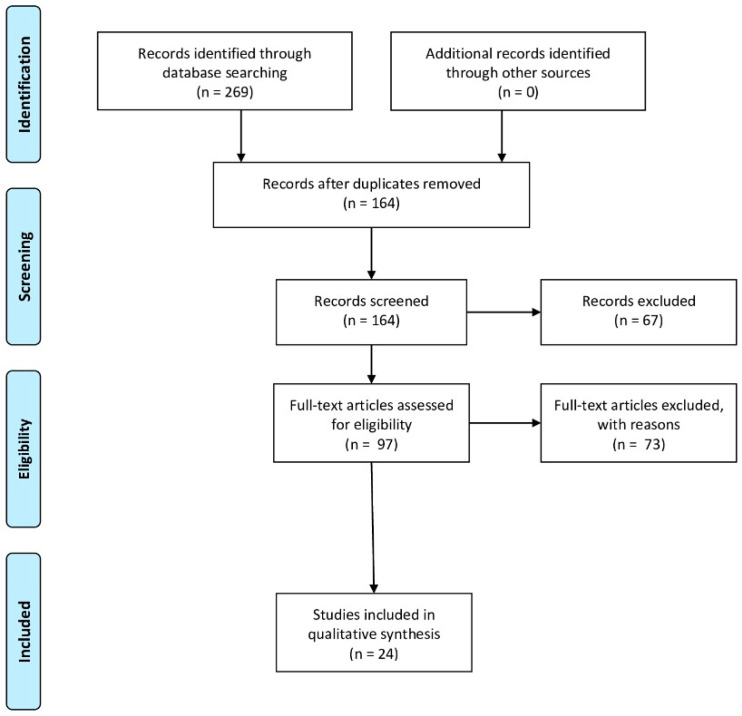
Prisma flow diagram illustrating the selection methodology of the preclinical studies used for the writing of the review. Duplicate articles were excluded from the total of the studies recorded. Instead, were considered articles that evaluate the damage of oxidative stress induced by fetal hypoxia (The PRISMA Statement was published in: [17]).

In order to write the paragraph “4. Animal models of prenatal hypoxia”, the bibliography research in PubMed was performed using the following keywords: “fetal hypoxia”, “prenatal hypoxia”, “placental hypoxia”, “oxidative stress”, “mice”, “rats”, “pigs”, “rabbits”, “antioxidants”, “neuroprotection”. In this review the articles published from 2000 to 2020 were taken into consideration. In this way, 269 articles were found, as shown in the Prisma flow diagram (Figure 1). In this manuscript were considered the preclinical studies evaluating the role of oxidative stress induced by prenatal hypoxia on the fetus were described. Moreover, in order to write the subparagraph “4.1. New antioxidant treatments for prenatal hypoxia” were also taken into consideration preclinical studies evaluating the effectiveness of new antioxidant agents.

## 3. Prenatal Oxidative Stress

Prenatal hypoxia is a condition responsible for the disease and fetal death or newborn [18]. The placenta is an organ important for communication between the pregnant woman and the fetus. The proper functioning of this organ is important for fetal development. Hypoxia is defined as a decrease in O_2_ necessary for the physiological functions of tissue [19]. An increase of ROS levels, generated from an incomplete reduction of O_2_, is one of the most common mechanisms induced to hypoxia. Normally the placenta produces ROS such as superoxide anion (O_2_^−^), hydroxyl radical (HO^−^) and hydrogen peroxide (H_2_O_2_) [20]. These molecules are highly unstable and possess a strong chemical reactivity due to the presence of unpaired electrons in the external orbital [21]. Due to this instability, ROS are inclined to yield or acquire an electron from other electrically unstable molecules, in order to achieve a stable energy state. In this way their lead a series of redox reactions that are important for the survival cellular. Normal ROS production is ensured by a balance between the production of these molecules and the antioxidant defense system. The main antioxidant system is provided to the activity of antioxidant enzymes such as superoxide dismutase (SOD), glutathione peroxidase (GPx) and catalase (CAT). Non-enzymatic antioxidants such as thiols (e.g., glutathione, GSH), protein thiols; vitamins A, B6, B12, C and E; selenium; folic acid; and the β-carotenoids, bilirubin and uric acid, represent another defense mechanism able to reduce the excessive ROS production [22]. During pregnancy, normal ROS levels can be involved in trophoblast proliferation and differentiation and in the modulation of the vascular responses of the placenta [19]. However, an increase in ROS levels is responsible for placental functional changes. Following fetal hypoxia, low levels of O_2_ lead to a reduction in the activity of the mitochondrial electron transport chain. Thereby, this reduction promoting an increase in the percentage of O_2_ incompletely reduced with consequent production of ROS, such as O_2_^−^ [20,23]. The mitochondrial electron transport chain represents a major ROS producer. Another source of ROS is represented by NADPH oxidase, responsible for the endogenous production of O_2_^−^ [24]. In the vascular endothelium, cytochrome P450 is another enzyme responsible for the production of OH^−^ and O_2_^−^ [25]. The metalloflavoprotein xanthine oxidase is another enzyme that following the oxidation of hypoxanthine to xanthine and uric acid, leads to the production of O_2_^−^ [26]. In the placenta, in hypoxic conditions, the mitochondrial oxygen consumption is downregulated. This leads to a reduction in the reserves of high energy phosphates generating high levels of xanthine, hypoxanthine, NADH, FADH, hydrogen ions (H^+^) and lactic acid [27].

Hypoxia induces a decrease in the enzymatic activity of the pumps of ATP-asi dependent membranes, reduction of the membrane potential and an increase in the flow of cytosolic calcium (Ca^2+^) levels. In hypoxic conditions, the increase in intracellular Ca^2+^, due to the activation of voltage-dependent channels and release by the mitochondria and the endoplasmic reticulum, establishes a loop that triggers the mechanism of apoptosis and neuronal necrosis [28]. Especially in the neuronal cells, the entry of Ca^2+^ favors the accumulation of glutamate. Glutamate, interacting with N-methyl-D aspartate (NMDA) receptors, intensifies the intracellular current of Ca^2+^, further contributing to neuronal damage [29].

Moreover, Ca^2+^ is responsible for activating nitric oxide synthase (NOS), involved in the production of nitric oxide (NO). Among the three known NOS isoforms, endothelial nitric oxide synthase (eNOS) is a Ca^2+^-dependent flavoenzyme that generates NO [30]. In this process Ca^2+^ plays an important role in the activation of eNOS, regulating the binding of eNOS with calmodulin [31] NO is a powerful endothelial vasodilator involved in the regulation of vascular tone, in the control of blood flow in the tissues and in the aggregation of platelets. In the placental, NO plays a key role in vasodilatation of the uteroplacental arteries, an important mechanism that determines the invasion of the trophoblasts and the remodeling of the endothelium [32]. Therefore, the altered balance of NO and ROS play a critical role in modulating the umbilical-placental vascular function in different prenatal conditions.

Therefore, high levels of ROS are responsible for damage to several cellular components such as DNA, proteins and lipids with consequent impairment of normal cellular functions [33]. During pregnancy, within 10–12 weeks of gestation, there is an increase in the flow of maternal blood into the placenta, which leads to a local increase in oxygen and consequently an increase in the activity of antioxidant enzymes. However, an excessive increase in ROS that cannot be countered by the antioxidant response, induce oxidative stress conditions. In the placenta oxidative stress, especially in this stage of pregnancy, is responsible for reducing the invasion of trophoblasts. This cascade of events induces different conditions that can be linked to alterations of fetal development and in serious cases even to early pregnancy failure [34,35].

The brain is more sensitive to changes in O_2_ levels. Oxidative stress is the main factor that induces neuronal cell death in the immature brain [36]. During embryogenesis, hypoxic damage also delays neuronal migration and alters the expression of numerous neurotransmitters [37,38]. These mechanisms increase the risk of neural birth defects, brain damage and long-term cognitive impairment in learning and memory [39,40]. Moreover, it can also predispose offspring to the future onset of epileptic conditions [41].

## 4. Animal Models of Prenatal Hypoxia

Several studies evaluate the effects of oxidative stress induced by prenatal hypoxia on the development of the fetus and the future consequences on offspring.

Matheson H. et al. have evaluated the chronic hypoxia in placental development. The authors explained about a reduction in fetal weight and an increase in placental weight due to hypoxic conditions by the Akt-mTOR signal and increased expression of heat shock protein 70 (HSP70) and HSP27, protecting the placentas from damage. Additionally, the authors reported a different gender-specific placental response to fetal hypoxia. They showed that the placentas of female fetuses were heavier and more sensitive to hypoxia-induced oxidative stress than those of males, although the mechanisms should be better clarified [42]. This finding was also supported by Song H. et al. in a model of pregnant guinea pigs and they demonstrated that chronic hypoxia inhibits mitochondrial function in placental tissues via increased peroxynitrite. This mechanism thus justifying the reduction of placental efficiency and fetal growth. Even in this study, the placental response exhibiting sexual dimorphism [43].

In order to clarify the role of oxidative stress in the prenatal hypoxia, Rueda-Clausen C. et al. used mice lacking the eNOS (eNOS^−/−^) and mice deficient in the enzyme catechol-O-methyl transferase (COMT^−/−^). Fetal hypoxia has led to a high mortality rate in eNOS^−/−^ fetuses compared to COMT^−/−^. Instead, was recorded an increase of NO in the placentas of COMT^−/−^ compare to eNOS^−/−^. This result would justify the increased tolerance to hypoxic stress in COMT^−/−^ mice. However, this hypothesis should be investigated further in order to enhance the understanding of placental responses to hypoxic insult [44].

Other studies focused in the role of oxidative stress induced by fetal hypoxia in the embryonal development of the brain in the offspring. Baud O. et al., proved that the brain of the offspring exposed to fetal hypoxia presented of cysts and extracellular matrix abnormalities. These results were justified with an increase in the activity of lipid peroxidation, presence of macrophages and consequent neuronal death. Thereby, chronic fetal hypoxia, through an increase of oxidative stress, may be the main cause of neurological impairment [45]. Instead, Sab I. et al. evaluated the involvement of fetal hypoxic damage in behavioral abnormalities and long-term disturbances in the learning and memory of offspring. In the offspring of 3-months exposed to prenatal hypoxia, the authors showed a reduction in exploratory capacity and locomotor activity and an increase in anxious behavior through a reduction in the activity of GPx and an increase in NO. However, would be necessary to better clarify the role of fetal damage-induced oxidative stress in the long-term disturbances in learning and memory in the offspring [40].

Prenatal hypoxia is also responsible of the fetal cardiovascular dysfunctions. Zhu X. et al. proven that fetal hypoxia induced a decrease in the partial pressure of O_2_ and the saturation of O_2_, in the fetal thoracic aortas. These results were correlated with an increase in response to angiotensin II (Ang II) an increase of O_2_^−^, due to activation of NADPH oxidase 4 and reduction of SOD [46]. Instead, Chen L. et al. wanted to evaluate the effects of maternal exposure to chronic intermittent hypoxia (CIH). In fetal hearts, the hypoxic conditions induced hypertrophy, high contractility of the left ventricle associated with high arterial stiffness. The authors correlated these cardiovascular abnormalities with a significant increase of lipid peroxidation and SOD1. Furthermore, was observed a major increase of lipid peroxidation in male offspring compared to that female [47]. The mechanisms underlying sex-dependent programming for offspring in pregnancy need further studies. Moreover, Figueroa H. et al., using New Zealand rabbits exposed to fetal hypoxia, showed an increased dilation of the vessels in fetal hearts. The authors justified this results with an increase in the activity of eNOS, inducible nitric oxide synthase (iNOS) and of the levels of carbonyl proteins, suggesting that oxidative stress is an important stimulus to initiate adverse effects in fetal hearts [48].

Other researchers focused attention in the alteration programming cardiovascular function induced by fetal hypoxia in adult offspring. In this regard, Giussani D.A. et al. proven that fetal hypoxia induced an aortic thickening in the fetus correlated with an increase in HSP70 and nitrotyrosine. Moreover, in the adult offspring (4 months) were also observed an increase in myocardial contractility and an alteration in the peripheral resistance vessels eNOS-dependent [49]. Instead Chen X. et al. compared the effects of prenatal hypoxia between 5-month and 20-month offspring. The authors showed vasodilation in the mesenteric arteries only in the 20-month offspring. These results were correlated with a decrease in eNOS activity and the antioxidants levels such as SOD and CAT. Moreover, was also observed an increase in ROS and malondialdehyde levels in both plasma and mesenteric arteries. This study has identified aging, as a postnatal factor that improves vascular dysfunction induced by fetal hypoxia [50]. In line with these results, Rueda-Clausen C. et al. demonstrated that the 12-month-old offspring exposed to chronic fetal hypoxia, shown high levels of total GSH. Additionally, in the male offspring, these events were responsible for the presence of multiple isolated areas of intra-ventricular myocyte death [51].

Furthermore, cardiovascular dysfunctions are also induced by epigenetic modifications due to altered ROS levels following fetal hypoxia [52,53]. Patterson A.J. et al. showed that hypoxic damage induced an increase of ROS responsible of epigenetic repression of the *PKCε* gene, in the hearts of rat offspring. This epigenetic mutation compromises the cardioprotective role of the protein encoded by *PKCε*. In this way, hypoxia-mediated ROS production in the developing heart is involved in the susceptibility of offspring to heart disease [54].

However, fetal hypoxia promotes damage also in the reproductive activity of the offspring. In this contest, Aiken C.E.et al. showed that hypoxic damage during the intrauterine period inducing aging oviducts in the 4-month female rats. These data were justified with increased markers of cell cycle such as p21 and p53 in the oviducts, two important regulators of the cell cycle. Parallelly, the oxidative stress negatively influences the functionality of the oviducts through alteration of the biogenesis of mitochondrial DNA [55]. Moreover, the authors in a later job reported that the offspring, shown a reduction in the number of primordial follicles available and an increase in oxidative stress markers such as *Gp91^phox^* and *P22^phox^*. Therefore, prolonged exposure to fetal hypoxia causes a reduction in fertility that accelerates with the aging of the ovary [56]. However, an understanding of these molecular pathways would be necessary to develop effective interventions to protect the female offspring of a high-risk pregnancy.

The results of all these studies showed that fetal hypoxia and the consequent increase in oxidative stress cause immediate adverse events on the fetus such as the limitation of fetal growth and the reduction of the weight of the unborn child. However, fetal hypoxia is also responsible for long-term damage to the offspring, such as an increased risk of subsequent cardiovascular dysfunction, cognitive delay and impaired reproductive function (Table 1).

### New Antioxidant Treatments for Prenatal Hypoxia

Complications of pregnancy such as fetal hypoxia activate physiological survival processes such as ROS production; therefore, clinical strategies such as the use of antioxidants can be helpful in managing fetal problems as well as long-term consequences on offspring (Table 2).

Okatani Y. et al. studied the effects of melatonin in improving the oxidative stress damage induced by ischemia/reperfusion of mitochondria in the rat placenta. Melatonin is an important scavenger secreted by the pineal gland that showed broad antioxidant, anti-inflammatory and antiapoptotic effects. The results showed that the administration of melatonin induced an increase in the markers of mitochondrial activity and a decrease in the concentration of reactive substances with thiobarbituric acid. Therefore, exogenous melatonin exhibited an antioxidant action and could be useful as a treatment for and fetal hypoxia [57].

Vitamin C is another antioxidant that may be used as a possible therapeutic treatment during fetal hypoxia. Vitamin C in mammals is one of the most important endogenous antioxidants. Richter H.G. et al. showed that maternal treatment with vitamin C prevented placental oxidative stress associated with maternal exposure to hypoxia [58].

Fetal hypoxia makes the offspring susceptible to the development of metabolic and cardiovascular disorders [59]. Resveratrol is a natural polyphenol that carries out its cardio-protective effects reducing ROS production, through the activation of molecules such as Adenosine Monophosphate Kinase Cardiac (AMPK) and the up-regulation of antioxidant enzymes such as SOD in endothelial cells of the arteries and smooth muscle cells [60,61]. Shah A. et al. evaluated the beneficial action of resveratrol in preventing alterations of metabolism and cardiac dysfunctions. The study showed that at 12 months of age, fetal hypoxia and the fatty acid diet have induced metabolic alteration in offspring, especially in male offspring. Resveratrol treatment has proven effective in preventing cardiovascular disease, probably through increasing cardiac SOD [62]. However, as in the previous study [62], even after 21 weeks, resveratrol treatment promotes an improvement in diastolic function and increases the ability to recover after an ischemia/reperfusion injury. The cardiovascular beneficial effects of resveratrol can be explained with a significant increase of the cardiac p-AMPK protein levels. Therefore, resveratrol could be a therapeutic opportunity used to counteract also the long-term oxidative damage induced by fetal hypoxia [63].

The use of mitochondrial antioxidants, such as MitoQ, could prevent the secretion of factors that cause DNA damage. Tom J. Phillips et al. assessed the ability of MitoQ to prevent DNA damage mediated by the release of harmful particles from the placenta induced following prenatal hypoxia. In order to prevent MitoQ from crossing the placenta, MitoQ was bonded with nanoparticles (nMitoQ), which accumulate in bilayered trophoblast barrier and do not cross through it, as previously demonstrated [64,65]. Administration of nMitoQQ appears to normalize alterations of microRNA, bone morphogenetic proteins and amino acids in placental secretions and plasma is capable of preventing molecular changes induced by fetal hypoxia, reducing oxidative stress in the placenta [66]. The same research team also evaluated the effects of placental treatment with nMitoQ in reducing the risk of developing cardiovascular disease in offspring exposed to prenatal hypoxia. The results of the study showed that hypoxia-induced changes in heart function that occurred following adulthood were attenuated by treatment with nMitoQ [67]. Additionally, the administration of nMitoQ reduced the O_2_^−^ levels in both the placenta and the fetus and the nitrotyrosine levels in the placenta. In this way, nMitoQ reduces the oxidative stress levels, highlighting that the efficacy of the treatment is targeted to the placenta. Moreover, treatment with nMitoQ resulted in an increase of vascular endothelial growth factor A (VEGFA) and insulin-like growth factor 2 (IGF-2). In conclusion, nMitoQ, reducing oxidative stress, improves oxygenation, angiogenesis and placental morphology, especially in the placenta of female offspring [68].

During the first week after birth, negative geotropism is important, both for rats and mice, to develop a sense of adaptation to the environment; just as straightening reflex is a neuromuscular response aimed at bringing the body into the normal vertical position [69,70]. Liu W. et al. observed that offspring subjected to hypoxic damage showed an alteration of these sensory responses. However, treatment with hydrogen during pregnancy restoring the anomalies of sensory responses and therefore preventing neurological damage induced by hypoxia [71].

Fetal hypoxia is also responsible for the alteration of neurological development and consequent cognitive delays, behavioral deficits, cerebral paralysis and other complications [72]. The erythropoietin shows neuroprotective properties [73] through several mechanisms of action including the reduction of oxidative stress [74] and damage induced by NO [75]. In this regard, Mazur M. et al. have proven that the administration of neonatal endogenous erythropoietin, 4 days later to hypoxic damage, has promoted the survival of oligodendrocytes and neurons. Additionally, improving histological damage was also observed, even after 24 days of treatment. In conclusion, erythropoietin improved the neurological insult induced by hypoxia and allow the correct development of the nervous system [76]. In view of these findings, Jantzi L.L. et al. assessed the effects of erythropoietin on calpain activated following fetal hypoxia. Calpain is a protein involved in cellular homeostasis during the development of the central nervous system and in the degradation of proteins in the mature central nervous system. Therefore, its alteration may be responsible for the cognitive delay [77]. The hypoxic damage induced high calpain activity postnatal in the cortex. The erythropoietin treatment reduced cortical calpain activity and promoted positive modulation of markers of neurological development, such as neuronal potassium-chloride co-transporter (KCC2), Myelin Basic Protein (MBP) and phosphorylated-neurofilament (p-NF). Thereby, erythropoietin can be valid therapeutic tools aimed at protecting the offspring from possible alterations in the development of the central nervous system [78].

## 5. Intrauterine Hypoxia and Human Evidence of Fetal Developing and Programming

The antioxidant defenses in the mother and placenta during fetal life and in the newborn born at term, under physiologic conditions, are capable of neutralizing ROS thus avoiding negative health consequences [79]. Increased production of ROS and oxidative stress during organogenesis, a period in which cells continue to differentiate, act as teratogenic agents; through disrupting critical signaling events causing structural abnormalities, loss of cellular function or spontaneous abortion of the developing fetus [79,80]. The relationship between oxidative stress and congenital malformations is not fully elucidated; however, oxidative stress may play a significant causal role in birth defects.

The prevalence of structural birth defects varies globally, ranging from approximately 3% to 6% of all live births [81]. These birth defects are a leading cause of infant mortality.

Most structural birth defects develop early in embryogenesis, during the first 10 weeks gestation, and the vast majority of birth defects are “nonsyndromic” and rare, not associated with multi-organ syndromes. A complex interaction between genes and environmental factors has been described [82].

For instance, the incidence of Congenital Heart Diseases (CHDs) varies from 4/1000 to 50/1000 live births [83] and only 15% of CHDs can be attributed to a genetic cause.

All other cases result from a complex interaction between genetic susceptibility and environmental factors (maternal drugs and alcohol assumption, nutrient availability, cigarette smoking, diabetic pregnancy, exposure to industrial chemicals, infectious agents) whose common embryotoxic effect may be related to oxidant-or redox misregulation with increased ROS production and alteration of developmental signals [83]. In particular, significantly lower concentrations of GSH and significantly higher concentrations of glutathione disulfide (GSSG) has been described [84,85,86].

In the same way the human brain is particularly vulnerable to the damaging effects of reactive oxygen intermediates due to both its complexity and the long period of development. Several studies to delineate the mechanism underlying maternal diabetic embryopathy demonstrated that oxidative stress is a major contributor in neural tube defects (NTDs) and other malformation such as Holoprosencephaly (HPE) [87].

NTDs result from a failure of the neural tube to close during the fourth week of embryogenesis. NTDs are among the most common of human birth defects, with an overall prevalence of around 0.5–2/1000 pregnancies and frequently result in infant mortality or major health problems in surviving children [81,88,89]. Most human NTDs occur sporadically, with recurrences tending to fit a multifactorial polygenic or oligogenic pattern, rather than either dominant or recessive single gene inheritance with reduced penetrance. In addition to the mutation of the coding sequence, altered transcriptional regulation of these genes has the potential to cause NTDs. A consideration of potential causative factors in human NTDs should, therefore, account for the possibility of deregulation of redox signals [79,90,91]. A direct relationship between antioxidant enzymes and the development of the neural tube is reported. GPx, glutathione S-transferases (GSTs) and copper-zinc super-SOD enzymes are the most important protective systems in humans for NTDs. An impaired responsiveness of the antioxidant enzymes, such as CAT, SOD, GPx, GSTs and glutathione reductase (GR) that play an active role in the detoxification of hydrogen peroxidase, has crucial effects in oxygen-induced embryopathy and may result in neural-tube fails [92,93,94].

HPE is characterized by midline defects of the brain, facial and oral structures. It affects 1 in every 5000–10,000 live births [95]. Many cases of human HPE occur following fetal alcohol exposure or as a result of maternal diabetes both associated with elevated levels of ROS [96,97,98,99]. In other congenital malformations, such as urological and sex disorders, the role of oxidative stress-mediated by ROS has been also proposed [100,101,102,103].

In adverse pre- and perinatal conditions, oxidative stress can also predispose the newborn to a variety of health issues in later life [104]. The fetal programming concept suggests that the intrauterine environment to which a fetus is exposed can have a long-term impact on health after birth. Although the exact mechanisms of fetal programming have not yet been examined, the correlation between intrauterine stress and adverse effects in offspring has been confirmed for diseases such as metabolic dysfunction, type 2 diabetes, cardiovascular disease, neurological disorders, obesity [49,105,106,107,108,109,110,111].

Finally, increased activity of some important antioxidant enzymes (SOD1, CAT and GR) together with decreased GSH levels and higher levels of biomarkers of oxidative damage, such as protein carbonyls, malondialdehyde, allantoin or 8-hydroxydeoxyguanosine has been reported in chromosomal disorders, such as Down’s Syndrome (DS) [112,113,114,115,116,117,118,119,120,121]. In particular, a relationship between oxidative stress and DS clinical expression has been proposed [79].

## 6. Areas for Future Research

Several drugs with antioxidant properties, such as melatonin, vitamin C, resveratrol, nMitoQ, hydrogen and erythropoietin. These antioxidants exhibit potential beneficial effects in fetal hypoxia. Some of these substances, such as vitamin C and melatonin, are administered during the gestational period, while nMitoQ represents a treatment targeting the placenta. Instead, resveratrol and erythropoietin are used as postnatal treatments. Furthermore, the release of antioxidants through nanoparticles in the placenta may represent a new therapeutic approach that, having as a target exclusively the placenta, can avoid adverse effects on developing offspring. Future studies will be needed before validating the use of these antioxidant treatments in the routine clinical application.

Moreover, the microbiota profile and contaminants, provide a key interface between the mother and the fetal development in pregnancy [122]. The environmental role on the target tissue, including the placenta, fetus and neonate is not fully elucidated and is a target for investigating mechanisms of fetal programming [123].

The role of the endocrine receptor, such as glucocorticoid receptor isoforms, on the response to stress, has been proposed [124]. However, the potential role of these receptors in adapting to the maternal environment and regulating fetal growth is still to be explored in future research. Recently, the effect on the immune response, inflammation and oxidative stress of stem cells, such as Mesenchymal Stromal Cells (MSCs) has been reported also during pregnancy conditions [125]. Further experimental and clinical investigations are mandatory to define the emerging therapeutic potential of MSCs on the prevention of placental oxidative stress and/or treatment of prenatal hypoxia.

Furthermore, early detection of oxidative stress markers may be also considered as a crucial point for a better knowledge of the regulation of gene expression under hypoxic conditions, in order to identify new targets and possible treatments [126].

Epigenetic processes act in a cell-specific, temporally regulated manner to direct development, differentiation, organogenesis and related processes [127]. The hypoxia-induced epigenetic modifications may be implicated in fetal metabolic programming, congenital malformations and chronic diseases in future life [128]. It is hoped that future research will provide insights at the molecular level into these hypoxia-induced epigenetic mechanisms and clinical problems, in order to detect the development of preventive and intervention strategies to combat the epigenetic disorders and associated disease [129,130].

Progress in this area of research could benefit from integrated analyses combining knowledge gained from studies of human cohorts, animal models and cell systems, to promote an understanding of children’s health and illness.

## 7. Conclusions

This review provides an overview of animal models commonly used in order to evaluate the effects of oxidative stress induced by placental hypoxia on the development of the fetus. The in vivo models employed the exposure of pregnant animals to different concentrations of O_2_ in hypoxic chambers and the vascular clamping of the uterine artery. These animal models showed increased ROS levels induced by fetal hypoxia with consequent predispose the newborn to congenital malformation and/or pathological conditions in later life.

Additionally, we considered the efficacy of novel therapeutic approaches in preclinical studies. To date, the most used antioxidant agents are melatonin, vitamin C, resveratrol, hydrogen, erythropoietin, MitoQ. Specifically, the release of antioxidants through nanoparticles in the placenta may represent a new therapeutic approach that could avoid adverse effects on developing offspring.

## Figures and Tables

**Table 1:** Synthesis of the studies that evaluate the role of fetal hypoxia-induced oxidative stress in several animal models.

Animal Models	Sample Size	O_2_ Concentration	Main Message	Ref.
Pregnant C57BL/6 mice	–	21% vs. 16% or 13%	Following hypoxic damage, as a response to oxidative stress, the placenta activates mechanisms that ensure the growth and survival of the fetus. The female placentas are more sensitive to hypoxia damage than male ones.	[42]
Pregnant Dunkin Hartley guinea pigs	Control group (n = 14 animals) Hypoxia group (n = 14 animals)	21% vs. 10.5%	In guinea pig chronic hypoxia altered mitochondrial function inducing placental dysfunction during pregnancy, especially in the male placentas.	[43]
Pregnant C57BL/6 J control mice; eNOS^−/−^ mice; COMT^−/−^ mice	–	20.9% vs. 10.5%	NOS^−/−^ mice showed less tolerance to hypoxic insult compare to C57BL/6 J and COMT^−/−^ mice. Greater bioavailability of placental NO in COMT^−/−^ mice could be mediating increased protection to hypoxic insult.	[44]
Pregnant Sprague Dawley rats	–	21% vs. 10%	Following hypoxic damage, microglial cells are activated as a response to oxidative stress, this causes the development of cysts in the white matter and the delay of myelination in the brain of the offspring.	[45]
Pregnant Wistar rats	–	–	Following hypoxic damage, through occlusion of the maternal uterine artery for 45 min, alteration of the antioxidant system can lead to behavioral abnormalities and long-term disturbances in learning and memory in the offspring.	[40]
Pregnant Wistar rats	4 groups of 20 animals each	21% vs. 13%	The adult offspring, following to hypoxic damage, showed an increase in myocardial contractility due to a NO-dependent endothelial alteration in the peripheral resistance vessels, demonstrating that oxidative stress influences cardiovascular programming.	[49]
Pregnant Sprague Dawley rats	–	21% vs. 10.5%	Following fetal damage, an increase of ROS leads to an increase to Ang II with consequent enhancement of vascular contractility.	[46]
Pregnant Sprague Dawley rats	Control group (n = 9 animals) CIH group (n = 6 animals)	21% vs. 4–5%	The CIH induces a significant increase in lipid peroxide and myocardial SOD1 activity that are responsible for cardiovascular damage.	[47]
New Zealand white rabbits	Control group (n = 7 animals) Hypoxia group (n = 6 animals)	–	Restriction of uteroplacental circulation in rabbits increased NO-dependent enzymes causing an increase in oxidative stress with consequent damage to cardiac function in fetuses.	[48]
Pregnant Sprague Dawley rats	Control group (n = 15 animals) Hypoxia group (n = 14 animals)	21% vs. 10.5%	The hypoxia damage in 20 months offspring induced an increase of ROS and malondialdehyde; while reduced eNOS activity and antioxidant enzymes such as SOD and CAT. In this way, hypoxia-induced oxidative stress plays a key role in vascular dysfunction. Additionally, aging is a postnatal factor, that promotes vascular dysfunction.	[50]
Pregnant Sprague Dawley rats	Control group (n = 16 animals) Hypoxia group (n = 16 animals)	21% vs. 12%	Fetal hypoxia causes long-term damage through increases in oxidative stress levels in the post-ischemic myocardium of adult offspring.	[51]
Pregnant Sprague Dawley rats	–	21% vs. 10.5%	Hypoxia-mediated ROS production in the developing heart expose the offspring to heart disease.	[54]
Wistar rats	Control group (n = 8 animals) Hypoxia group (n = 8 animals)	21% vs. 13%	Fetal hypoxia can also be responsible for the accelerated aging of the oviducts, thus compromising the reproductive function of potential mothers.	[55]
Wistar rats	Control group (n = 8 animals) Hypoxia group (n = 8 animals)	21% vs. 13%	Fetal hypoxia in addition to accelerated aging of the ovary is also responsible for a reduction in fertility through an increase of oxidative stress.	[56]

eNOS; endothelial nitric oxide synthase, eNOS^−/−^ mice; mice lacking the eNOS, COMT^−/−^ mice; mice deficient in the enzyme catechol-O-methyl transferase, NO; nitric oxide, Ang II; angiotensin II, CIH; chronic intermittent hypoxia, SOD1; superoxide dismutase1, ROS; reactive oxygen species, CAT; catalase.

**Table 2:** Synthesis of the studies aimed at testing the antioxidant properties of some substances such as melatonin, vitamin C, resveratrol, nMitoQ, hydrogen and erythropoietin. Specifically, the table shows the animal models used in the studies and the type of hypoxic damage induced. Additionally, the type of treatment, the dosage, the route of administration and the therapeutic effects obtained are described.

Antioxidant Treatments	Animal Models	Hypoxic Damage	Treatment	Dosage	Route of Administration	Therapeutic Effects	Ref.
MELATONIN	Pregnant Wistar rats	Occlusion of the uterine artery for 20 min; GD 15.	Prenatal (1 h prior to fetal hypoxia)	10 mg/kg	Intraperitoneal injections	Reduction of ROS	[57]
VITAMIN C	Pregnant Wistar rats	Hypoxic conditions (13% O_2_); GD 6–20.	Prenatal (every day during pregnancy)	5 mg/mL	Drinking water	Prevention of oxidative damage; improvement of placental function and protection fetal growth.	[58]
RESVERATROL	Pregnant Sprague Dawley rats	Hypoxic conditions (11% O_2_); GD 15–21.	Post-natal (for 9 weeks)	4 g/kg	Diet integration	Promotion of cardiac recovery by increasing cardiac SOD.	[62]
			Post-natal (for 18 weeks)			Reduce heart damage by increasing in cardiac p-AMPK and SOD2 levels.	[63]
nMITOQ	Pregnant Sprague Dawley rats	Hypoxic conditions (11% O_2_); GD 16–21.	Placental (GD 15)	125 μM	Intravenous injections	Restoration of molecular changes induced by fetal hypoxia such as microRNA, bone morphogenetic protein and amino acids and reduction of oxidative stress in the placenta.	[66]
						Improvement of the sensitivity to vasorelaxation and the systolic dysfunction in the offspring of 7 and 13 months and reduction of placental oxidative stress.	[67]
						Improvement of the oxygenation, angiogenesis and placental morphology, especially in the placenta of female offspring.	[68]
HYDROGEN	Pregnant Sprague Dawley rats	Hypoxic conditions (8% O_2_% and 92% N_2_); GD 17–18.	Prenatal (4 h of exposure to this condition at GD 17–1 at the term)	Mixture of hydrogen (2% H_2_, 8% O_2_% and 90% N_2_)	–	Restoration of the anomalies of sensory responses and prevent neurological damage induced by fetal hypoxia.	[71]
ERYTHROPOIETIN	Pregnant Sprague Dawley rats	Occlusion of the uterine artery for 60 min; GD 18	Post-natal (After 4 days from the fetal hypoxia per 5 days; PD 1–5)	500 U/kg per 1 day, 1000 U/kg per 3 days and 2000 U/kg per 5 days.	Intraperitoneal injections	Improvement of the neurological damage and the correct development of the nervous system.	[76]
				2000 U/kg		Reduction of the excessive activity of the calpain and protection of the central nervous system.	[78]

GD; gestational days, PD; post-natal days, ROS; reactive oxygen species, SOD; superoxide dismutase, NAC; N-acetylcysteine, AMPK; adenosine monophosphate kinase Cardiac.
